# The Five Diaphragms in Osteopathic Manipulative Medicine: Neurological Relationships, Part 1

**DOI:** 10.7759/cureus.8697

**Published:** 2020-06-19

**Authors:** Bruno Bordoni

**Affiliations:** 1 Physical Medicine and Rehabilitation, Foundation Don Carlo Gnocchi, Milan, ITA

**Keywords:** diaphragm, osteopathic, fascia, myofascial, fascintegrity, physiotherapy

## Abstract

In osteopathic manual medicine (OMM), there are several approaches for patient assessment and treatment. One of these is the five diaphragm model (tentorium cerebelli, tongue, thoracic outlet, diaphragm, and pelvic floor), whose foundations are part of another historical model: respiratory-circulatory. The myofascial continuity, anterior and posterior, supports the notion the human body cannot be divided into segments but is a continuum of matter, fluids, and emotions. In this first part, the neurological relationships of the tentorium cerebelli and the lingual muscle complex will be highlighted, underlining the complex interactions and anastomoses, through the most current scientific data and an accurate review of the topic. In the second part, I will describe the neurological relationships of the thoracic outlet, the respiratory diaphragm and the pelvic floor, with clinical reflections. In literature, to my knowledge, it is the first time that the different neurological relationships of these anatomical segments have been discussed, highlighting the constant neurological continuity of the five diaphragms.

## Introduction and background

In osteopathic manual medicine (OMM), five models are recognized for the patient's management and clinical setting: behavioural, biomechanical, neurologic, metabolic, and respiratory-circulatory [1]. The clinician can adopt a modular approach or move towards a strategy that involves all models. The latter choice should represent the most appropriate approach for a complete involvement of the patient's well-being, as the human body cannot be divided into segments but is a continuum of matter, fluids, and emotions [2,3]. The founder of osteopathy, Dr Andrew Taylor Still, in 1874 described a fundamental concept for OMM, that is, that an injury arises when there is an interruption of fluids; this event can materialize as a somatic, visceral, and behavioural symptom [4]. The five diaphragms are part of the respiratory-circulatory model, where the latter's philosophy is based on the optimal circulation of body fluids through functional relationships between some segments of the body considered as diaphragms: tentorium cerebelli, tongue, thoracic outlet, diaphragm, and pelvic floor [5,6]. The nourishment and cleansing of all tissues by the blood and lymph, as well as their free circulation are fundamental for physical and mental health. If the tissues are free to move and are able to slide one over the other, as well as at the cellular level, body fluids can perform countless functions and transport multiple substances. If the blood flow to the different tissues is adequate, for example, the insulin produced by the pancreas can improve the function of the central and peripheral nervous tissue. Insulin acts at the level of its receptors (InsRs), which are part of the family of receptor tyrosine kinase, with a heterotetrameric structure and four glycoproteins (two of the extracellular alpha type and two of the transmembrane beta type) [7]. The hormone activates the InsRs at the level of the peripheral nerves (on the axolemma) influencing the function of the myofascial tissues (and the viscera), as it allows a correct cycle of repair and tissue adaptation, as well as of the nervous tissue itself; insulin also modulates somatic and visceral nociceptive afferents [7]. The activation of InsRs activates a metabolic cascade involving the stimulation of phosphatidylinositol-3-kinase (PI3K), some kinase proteins (PKC and Akt), growth factors and in particular, activates the action of mitogen-activated protein kinase (MAPK); the latter is essential for the development and regeneration of the tissue and for keeping the ribonucleic acid messenger (mRNA) stable [7]. Another example comes from the possibility of retrograde movement of exosomes. The latter are vesicles released by different types of cells and are a communication tool between cells and cell or for very distant cells [8]. The peripheral endings at the myofascial level can send exosomes which, retrogradely go up the axon to get to the medullary neuron, influencing its behaviour or favouring neural regeneration and adaptation [8]. This happens thanks to the transport and subsequent release of information, such as micro ribonucleic acid messengers (miRNAs); miRNAs are capable of modulating epigenetic phenomena [9]. Only thanks to the free movement of fluids are miRNAs able to move. The presence of movement of the fluids indicates greater available space and this is another reason (space) for which multiple substances can express their functions at best [10]. The lymph itself carries exosomes to correctly influence the systemic immune response [11]. To further underline the importance of the circulation of fluids and to corroborate the model of the five diaphragms, is the relationship between blood and the psychological aspect. Red blood cells or erythrocytes can be considered as endocrine glands capable of secreting many substances [12]. Among the substances produced in paracrine mode, there are some molecules that result from the degradation of haemoglobin, through enzymes such as proteasomes and oligopeptidases. From this degradation, we find hemopressin (Hp) and hemophin, which attach to cannabinoid receptors (CBr1 and CBr2) [12]. A portion of hemopressin, in particular, Hb-alpha1 can be considered a neuropeptide, capable of having anti-nociceptive effects (reduces the sensations of hyperalgesia and visceral pain), is capable of improving memory and ability to regulate the mood [13]. Hp stimulates the release of endorphins, exerting anxiolytic and antidepressant effects as allosteric agents, targeting CBr1 and CBr2 centrally [12,13]. Hemophin would act to promote a correct anti-inflammatory and anti-nociceptive response, always through the endocannabinoid pathway [12,13]. Body diaphragms must be considered as three-dimensional structures, represented on multiple planes and axes; it is a mistake to consider a body structure extrapolated from its three-dimensionality. The wide technical background of the clinician who applies OMM can offer numerous manual approaches to diaphragms [14]. In this first part, the neurological connections of the tentorium cerebelli and the lingual complex will be described, highlighting the nerve interactions and anastomoses, through the most current scientific data and an accurate review of the topic. In the second part, I will deal with the neurological interactions of the remaining diaphragms (thoracic outlet, diaphragm, and pelvic floor) with clinical reflections.

## Review

Innervation of the tentorium cerebelli

**Figure 1:**
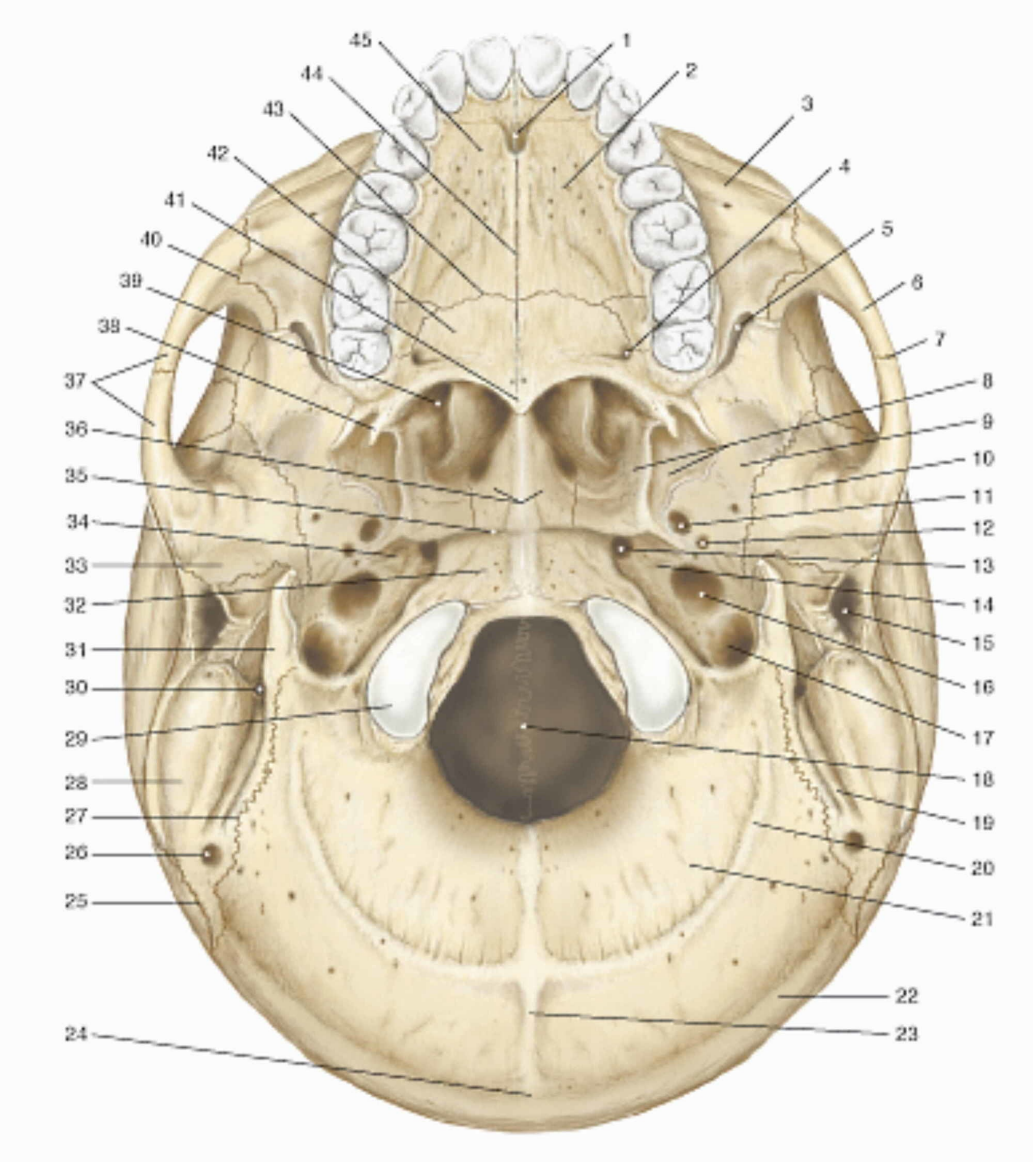
The figure illustrates the external surface of the skull base 1: incisive foramen; 2: maxillary bone; 3: zygomatic process of the maxilla; 4: greater foramen palatine; 5: lower orbital fissure; 6: zygomatic bone; 7: temporozygomatic suture; 8: pterygoid process; 9: great wing of the sphenoid; 10: sphenosquamosal suture; 11: foramen ovale; 12: foramen spinous; 13: lacerate foramen; 14: pyramid of the temporal; 15: external acoustic meatus; 16: carotid canal; 17: jugular foramen; 18: foramen magnum; 19: mastoid notch; 20: inferior nuchal line; 21: nuchal plane; 22: superior nuchal line; 23: external occipital crest; 24: inion; 25: lambdoid suture; 26: mastoid foramen; 27: occipitomastoid suture; 28: mastoid process; 29: occipital condyle; 30: stylus mastoid foramen; 31: styloid process of the temporal; 32: basal portion of the occiput; 33: mandibular fossa; 34: groove of auditory tube; 35: spheno-occipital synchondrosis; 36: wings of the vomer; 37: zygomatic arch; 38: pterygoid hook; 39: inferior nasal concha; 40: zygomatic maxillary suture; 41: inferior nasal spine; 42: horizontal lamina of the palatine; 43: transverse palatine suture; 44: median palatine suture; 45: palatine process of the maxilla. Reproduced with permission, from Anastasi G, et al., Anatomia dell'uomo, fourth edition [Human Anatomy], 2010, Milan: Edi-Ermes, Volume 1, p 105

The tentorium cerebelli is the dural portion where the falx cerebri (above) and the falx cerebelli (below) cross: the tentorium divides the brain from the cerebellum. For the clinician who performs the OMM, it can arise problem palpation, when the tentorium presents anatomical abnormalities, such as duplication, a triplication or a complete absence [15,16]. The upper portion of the tentorium cerebelli, in addition to possessing important characteristics of mechanical support for the central nervous system, also has a metabolic activity influenced by mechanical and electrical stimuli [17]. A branch of the ophthalmic nerve, the nervus tentorii innervates the supratentorial area with subjective orientations and arborisations (four types of direction/arborization classified) [17]. The innervation mainly involves the tentorial notch area and the portions of the straight sinus and transverse sinus; the nerve involves the middle meningeal artery and rarely the posterior portion of the falx cerebri [17]. The nervus tentorii innervation travels throughout the territory of the supratentorial area, with different density percentages. Occasionally, the latter nerve can innervate the lateral dural occipital area [17]. The dural afferent projections of the ophthalmic nerve/nervus tentorii end in the spinal cord area at the level of C2, in lamina V/VI of the dorsal horn, together with the afferents of the Arnold nerve (C2) [18]. The sympathetic and parasympathetic system involves the tentorium area: both the supratentorial area and the subtentorial area. Parasympathetic fibers for the supratentorial area probably originate from the sphenopalatine ganglion (to a lesser extent) and from the venous and arterial blood vessels involving the dura; a sympathetic branch of the vagus, which anastomoses with the sympathetic pathway that follows the middle meningeal artery, innervates the supratentorial area [18,19]. The sympathetic innervation of the supratentorial area derives from the middle meningeal artery, which artery carries the sympathetic pathways coming from the upper cervical sympathetic ganglion [20]. The sympathetic and parasympathetic afferents derive mainly from the dural vascular plexus, through type A myelinated, non-myelinated type C fibers, and free fibers (subtype); these afferents may concern Ruffini-like non-encapsulated receptors [18]. In their path, type A and C fibers can be anastomosed to converge in the spinal cord at the level of C1-C3; fibers A travel towards lamina III-V of the dorsal horn, while fibers C travel towards the dorsal medullary horn in lamina I-II [18]. The afferents of the first cervical roots and the projections of some portions of the spinal trigeminal nucleus (interpolaris and caudalis) will flow into this medullary area [18]. Autonomic afferents carry multiple information from mechanical (traction), thermal, chemical, blood pressure and pain stimulations, while trigeminal afferents (ophthalmic nerve/nervus tentorii) will mainly bring painful information [18]. Subtentorial innervation is more complex. If anteriorly, we find the ansa cervicalis with the ventral roots of the first cervical roots, posteriorly there is the plexus of Cruveilhier. The latter involves the first three dorsal cervical roots such as C1-C3 and more rarely C4; are the dorsal roots of C2-C3 which directly innervate the subtentorial area [21,22]. For other authors, the dorsal root of C1 also converges in the subtentorial area [18,23]. The dorsal root of C1, the greater occipital nerve (GON) or C2 and the lesser occipital nerve (LON) or C2-C3, together with branches of the sympathetic trunk and following, in particular, the blood tract, penetrate the skull [22,23]. The ways of entry into the skull can be: the foramen magnum; small holes in the skull following the venous vascular pathways such as those lateral to the occipital condyles; the hypoglossal canal and the jugular foramen (Figure 1) [22,23].

The C1 nerve is not always present and does not always have a ganglion; has a close relationship with the spinal dura, where, when the pierces the dura forms a sinuvertebral branch or Luschka branch [24]. The first three spinal nerves converge and have close relationships with the spinal trigeminal nucleus [25]. The subtentorial afferents of spinal origin, in particular GON, most likely, carry nociceptive information towards the medullary area of the dorsal horn to lamina V/VI [18,22]. The subtentorial area is innervated by some cranial nerves, such as recurrent branches of the vagus nerve (X), by meningeal nerve branches of the hypoglossal (XII), by branches of the glossopharyngeal nerve (IX); all these last cranial nerves in their path become anastomosed, with connections of neurological continuity [23]. We cannot exclude the possibility that some branches of the seventh cranial or facial nerve may innervate the subtentorial area, in admixture with parasympathetic filaments [23]. Another possibility of the neurological influence of the subtentorial portion could derive from the trochlear nerve or IV cranial nerve. The trochlear nerve, which according to the texts examined can be divided into four to six segments, has a specific tentorial segment; the latter has an average length of about 13.5 millimeters and passes under a section of the subtentorial area and in a dural canal [26]. In this segment, it receives several vascular branches, including the branches from the Bernasconi-Cassinari tentorial arteries [27]. Tentorium cerebelli can be the source of different forms of migraine when its structure (dura, vessels, and nerves) is altered [22].

Innervation of the lingual complex

The lingual complex is represented at the level of the central nervous system with a very specific somatotopic organization: somatosensory cortex, mesencephalon, medulla oblongata, and limbic system [28]. The tongue is innervated by multiple nerve branches. The hypoglossal nerve or cranial XII nerve can be divided into two portions: intracranial and extracranial and in five segments (cisternal, intracanalar, descending, horizontal, and ascending) [29]. The nucleus of the XII is placed behind the medulla oblongata and through a craniocaudal direction; it crosses the medulla oblongata, and then exits with some roots or branches, ending in the dural pore, thus forming the cisternal segment [29]. Before entering the dura mater, these branches envelop or touch the posterior inferior cerebellar artery and the vertebral artery [29]. When the nerve passes the dural pores and enters the hypoglossal canal, the intracanal segment begins. In the hypoglossal canal, we can find an arterial branch of the ascending pharyngeal artery and a venous plexus, which drains into the terminal portion of the sigmoid sinus and through small bone holes, drains towards the jugular bulb; the plexus probably derives from the anterior condylar vein. The hypoglossal canal is located laterally to the jugular foramen and to the jugular process of the occipital bone; the occipital condyles are placed inferiorly while a part of the sphenoid area (clivus) is superomedially [29]. Nerve XII is covered for two-thirds of its path in the canal by the dural sheath and the arachnoid sheath. When the nerve exits the canal, we find the descending segment, where it enters the neck, under the sternocleidomastoid muscle (SCM), in an anatomical area where we find the small rectus capitis, the internal carotid, the vagus nerve, the glossopharyngeal, and accessory nerve (medial to XII) [29]. Following the internal carotid and jugular vein, the nerve meets some muscles (stylopharyngeal, stylohyoid, styloglossus, digastric). Continuing in its path, the nerve meets the lateral portion of the external carotid artery and the anterior area of the SCM. The horizontal segment of the XII nerve begins passing between the greater horns of the hyoid bone, touching the tendon of the digastric muscle (the nerve passes under the tendon). In this area, we can find the submandibular gland, the upper cervical fascia and subcutaneous fat, the platysma and stylohyoid muscles laterally to the nerve; medially to the nerve, we can recognize the constrictor muscles of the pharynx and the hyoglossus [29]. In the horizontal segment, the nerve has a close relationship with the lingual artery. The ascending segment of the XII nerve starts from the edge of the mylohyoid muscle district, to reach the lower area of the tongue, the tip of the tongue and the dorsal area, following a path that places the nerve between the mylohyoid muscle (laterally) and the hyoglossus muscles and genioglossus (medially). The nerve comes into contact with the Wharton duct and the lingual artery [29]. The lingual nerve (LN) is a branch deriving from the mandibular branch of the trigeminal nerve, with a sensory setting. LN arises after the mandibular nerve passes through the oval foramen and giving branches such as the auriculotemporal nerve, the lower alveolar nerve, and the LN in the infratemporal fossa [30]. LN follows a descending path towards the medial edge of the lateral or external pterygoid muscle; before reaching the muscle, we find an anastomosis with the lower alveolar nerve, while before completing the path near the muscle, LN receives the chorda tympani nerve (VII cranial nerve) [30]. LN receives the nervus mylohyoideus (motor and sensory nerve) always deriving from the mandibular nerve; this connection should take place after anastomosis with the nerve VII [31]. LN continues its path between the medial or internal pterygoid muscle and the mandibular bone branch, follows an anteromedial direction towards the posterior insertion of the mylohyoid muscle. LN penetrates into the mouth below the lower margin of the constrictor muscle, in the area of the third molar (behind and under the tooth); contact the periosteum of the jaw and/or the upper area of the mylohyoid muscle. LN receives the parasympathetic nerve endings of the mandibular ganglion, which is located near the second molar [30]. LN continues in a lower direction towards the lower surface of the muscle or lingual complex and wraps the submandibular duct, finally reaching the front two-thirds of the tongue with its sensory endings [30]. The LN is able to transport the perception of mechanical stimuli, such as proprioception, to the trigeminal ganglion. The XII and the LN branch within the tongue. These two nerves form anastomoses in extra lingual areas, inside the tongue, in particular for the hypoglossal muscle and the apex of the tongue; the LN creates anastomosis with the contralateral LN at the tip of the tongue [32]. The glossopharyngeal cranial nerve or nerve IX, motor and sensory nerve and with parasympathetic components, innervates the posterior one-third area of the tongue [33]. The IX arises from the upper portion of the medulla oblongata by several filaments; it exits the cranial cavity through the jugular foramen, together with the vagus nerve and the accessory nerve (XI), passing anteriorly, respect to the X and XI [33]. In the foramen, it forms two ganglia: upper ganglion of the Ehrenritter or jugular, purely sensitive; lower or petrosal ganglion, with sensory/parasympathetic component [33]. The lower ganglion transmits the sensations of taste and stereognosis information from the tongue and mixed sensations from the oropharynx [33]. Once exited from the jugular foramen, the nerve runs downwards, between the internal carotid artery and the internal jugular vein; passes medially to the styloid process and the internal and external carotids performs a curvature anteriorly around the lower portion of the stylopharyngeus. Continue to pass between the middle pharyngeal constrictor muscle and medially to the hyoglossus/stylohyoid ligament and the posterior terminal portion of the tongue; from here, the IX enters the tongue, in particular, the stylopharyngeus muscle and the middle and upper constrictor muscles, and the epiglottis [33]. The nerve IX has several ramifications: it anastomoses with the vagus nerve (via a filament that connects the superior ganglion with the jugular ganglion), with the facial nerve (with a filament placed immediately below the petrosal ganglion) and with the upper cervical ganglion ( by means of a filament from the petrous ganglion); tympanic (Jacobson) nerve which is a contingent of parasympathetic (or visceral) sensitive fibers originating from the petrosal ganglion and parasympathetic effector fibers, which originate from the lower salivary nucleus, and then help to form the tympanic plexus. The parasympathetic fibers of the tympanic nerve first become part of the small superficial petrous nerve and the otic ganglion (Arnold), to finally distribute themselves to the parotid gland [33]. Other branches of the IX are the tonsillar branches (they come to innervate the soft palate, the palatine tonsils and the oropharyngeal isthmus), pharyngeal branches, and carotid branches, the latter descend to the carotid sinus and communicate with sympathetic nerves and branches of the vagus nerve. The IX anastomizes with its contralateral branches, with the XII and the LN and the X at the level of the tongue [34]. The facial nerve or VII arises from the pons in the facial nucleus (consisting of three nuclei: motor, sensory and parasympathetic) and has six segments; before penetrating the temporal bone (internal auditory meatus) it is reached by the nervus intermedius or Wrisberg; both are directed to the geniculate ganglion [35]. The VII provides multiple motor and sensory branches. Wrisberg's nerve provides sensory and parasympathetic information from the sublingual and submandibular salivary glands, as well as from the palatine and nasal glands; carries taste information from the anterior two-thirds area of the lingual muscle complex and from the soft palate [35]. These afferents that reach the brainstem involve the spinal trigeminal nucleus, the LN, nucleus of the tractus solitarius, the thalamus (medial lemniscus), insula and sensory cortex, the hypothalamus [30,35]. The sympathetic superior cervical ganglion innervates the tongue, in particular, the submucosa of the tongue; at the level of the posterior portion there are sympathetic fibers ipsilaterally and contralaterally [36]. The X cranial nerve and in particular the rostral area of the nucleus of the tractus solitarius receives the sensations of taste through the VII and through the IX nerve [19]. The lingual complex is involved in several cranial nerves and systems: VII, V, IX, X, XII, sympathetic system.

Pharyngeal plexus and ansa cervicalis

**Figure 2:**
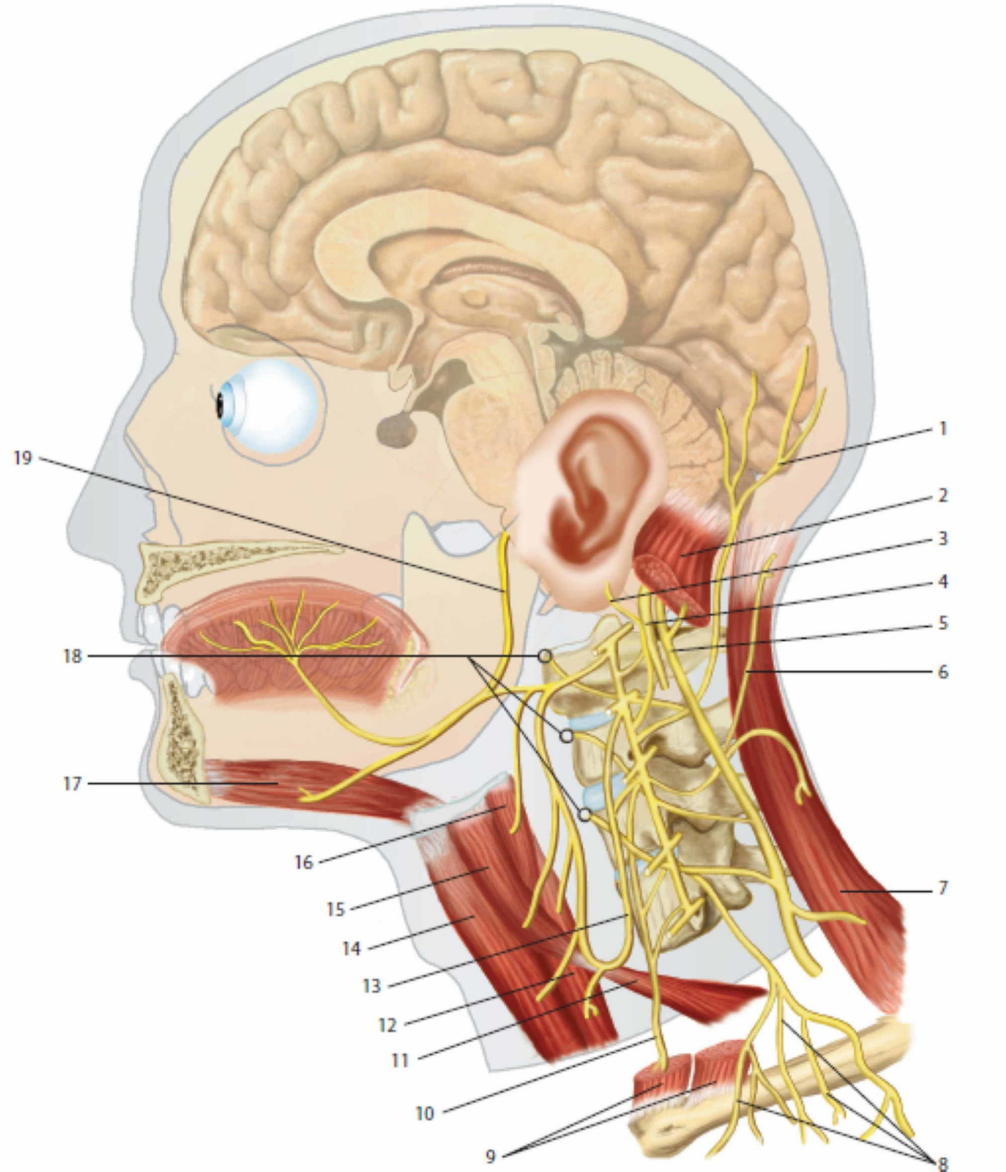
The figure illustrates the cervical plexus and the course of the skin and muscle branches; the relationships of the cervical plexus with the cranial nerves X, XI, XII are demonstrated 1: lesser occipital nerve; 2: sternocleidomastoid muscle; 3: greater auricular nerve; 4: cranial nerve X; 5: cranial nerve XI; 6: cutaneous nerve of the neck; 7: trapezius muscle; 8: supraclavicular nerves; 9: sternocleidomastoid muscle; 10: phrenic nerve; 11: homohyoid muscle (lower portion); 12: sternothyrohyoid muscle; 13: descending cervical nerve; 14: sternohyoid muscle; 15: homohyoid muscle (upper portion); 16: thyrohyoid muscle; 17: geniohyoid muscle; 18: prevertebral muscles; 19: cranial nerve XII. Reproduced with permission, from Anastasi G, et al., Anatomia dell’uomo, fourth edition [Human Anatomy], 2010, Milan: Edi-Ermes, Volume 3, p 247

The pharyngeal plexus (PP) is positioned in the posterior portion of the middle pharyngeal constrictor muscle; the plexus is formed by branches of the nerve IX, X and the sympathetic upper cervical ganglion [37]. The PP is found in the retropharyngeal space near the neck and longus capitis muscles, the connective tissue of the prevertebral fascia of C2-C3 (posteriorly), and the posterior edge of the pharynx. PP originates initially from the ambiguous nucleus (medulla oblongata), demonstrating a common origin with the vagus nerve or cranial X. We find the origin of the branches of the glossopharyngeal nerve or pharyngeal branches for the contribution to the PP from the inferior and superior petrous nuclei (inside the jugular foramen); the tonsillary branches of the IX in less percentage help the formation of the PP [37]. The pharyngeal and tonsillar branches of the IX have afferent sensory skills. The extracranial ganglion of the vagus or nodose nerve is important for the constitution of PP through sensory pharyngeal branches. The sympathetic superior cervical ganglion (SSG) found at the ventral level of the transverse process of the epistropheus or C2. SSG has three groups of bundles: the lateral branches connected with the first four cervical roots through communicating grey branches, the vagus, the glossopharyngeal lower ganglion and the T1 nerve; the medial branches known as cardiac and laryngopharyngeal branches connect with the superior laryngeal nerve and the ascending aortic nerve plexus; anterior rostral branches connect with the VII and the intracranial area [37]. PP connects with the intercarotid and carotid plexuses; for some authors, these plexuses could be a more lateral extension of the same PP. The IX and X communicate not only intracranially but extracranially through the inferior or petrosal ganglia or of Andersch (IX) and the jugular ganglion of the X; communicate through Haller's ansa, an anastomosis between the IX and X (Arnold branch or an auricular branch) and a branch of the VII. These anastomoses contribute to the formation of PP [37]. We can find many efferences and afferents to and from the PP. The efferences and afferents for swallowing are in particular the task of the vagal system (solitary tract nucleus, ambiguous and dorsal nuclei, nucleus of the XII). Other afferents run along the nerve IX involving the solitary tract nucleus in their path, which will send afferents to the trigeminal motor nucleus, the VII and the ambiguous nucleus and the XII [37]. PP connects the lingual complex and tentorium cerebelli indirectly. The ansa cervicalis (AC) is another structure that involves cervical roots and cranial nerves. We can divide the AC into two portions, of which an upper one consisting of the first two ventral cervical roots or only with the root of C1 and a lower portion with the ventral roots of C2-C3 and sometimes C4. [38]. AC is part of the cervical plexus with the main objective of innervating the sub-hyoid muscles. For a short distance, the ventral root of C1 anastomoses with the descending segment of the nerve XII for 3-4 centimeters; from here, branches of C1 descend along the front edge of the carotid sheath to form, together with the roots of C2, the upper portion of AC [39]. Animal studies show that the branches of C1-C2 that form the upper CA can provide other more distal connections with the XII, with motor and proprioceptive functions to the tongue [40]. C1 can have an anastomosis with the XI nerve or cranial accessory nerve and/or with vagal components; a small percentage of XII fibers could follow the C1 root [39,41]. Some ascending fibers of the AC of the upper portion could act as support nerves to the XII for subtentorial innervation or meningeal branches of the XII [41]. The AC has always descending and ascending fibers and this entails a multiplicity of connections [39-41]. The lower portion of the AC is formed by the ventral roots of C2-C3 and/or with some C4 fibers. The fibers with the lower ascending branches can be traced back until reaching the XII nerve [41]. Other descending branches can branch up to the thorax to anastomise with the cardiac nerves of the stellate sympathetic ganglion and the phrenic nerve; horizontal branches can anastomise with the sympathetic cervical trunk and with accessory phrenic nerves and/or the subclavian nerve [39]. The AC connects, directly and indirectly, the tentorium cerebelli, the tongue, the thoracic outlet and the respiratory diaphragm. From the AC, we can identify other interesting branches. The great auricular nerve is a cutaneous nerve that originates from fibers from C2 and C3. It innervates the skin of the parotid and mastoid regions and part of the auricle and the parotid fascia (the fibrous structure that lines the parotid gland in the parotid loggia). It emits branches that anastomize with the greater occipital nerve, the lesser occipital nerve and with the posterior auricular nerve of the VII nerve [42]. The transverse cervical nerve or transverse cutaneous nerve of the neck is a cutaneous nerve that originates from AC from fibers coming from C2 and C3; it is distributed to the skin of the supra-hyoid and sub-hyoid regions and creates an anastomosis with the greater auricular nerve [43]. There is a medullary connection with the fibers of the first cervical roots and the spinal trigeminal nucleus (STN), which is in close connection with the trigeminal ganglion of Gasser [44]. The C2 ganglion or dorsal root ganglion (DRG) sends fibers to the cochlear nucleus, where other fibers will arrive, such as from the STN; DRG also sends fibers directly to the spinal trigeminal nucleus [45]. Recall that the trigeminal sensory neuropathy (TSN) receives information from the many cranial nerves, such as the V, VII, IX, and X. The ventral branches of the first four cervical roots form the cervical plexus; the cutaneous branches innervate the sensitivity of the skin near the auricle (greater auricular nerve and in part the lesser occipital nerve), neck and clavicle (transverse cervical nerve and the supraclavicular nerve, respectively), while the motor branches particularly innervate the muscles sub-hyoid (except the thyrohyoid muscle) and the diaphragm muscle (phrenic nerve) (Figure 2) [46].

**Figure 3:**
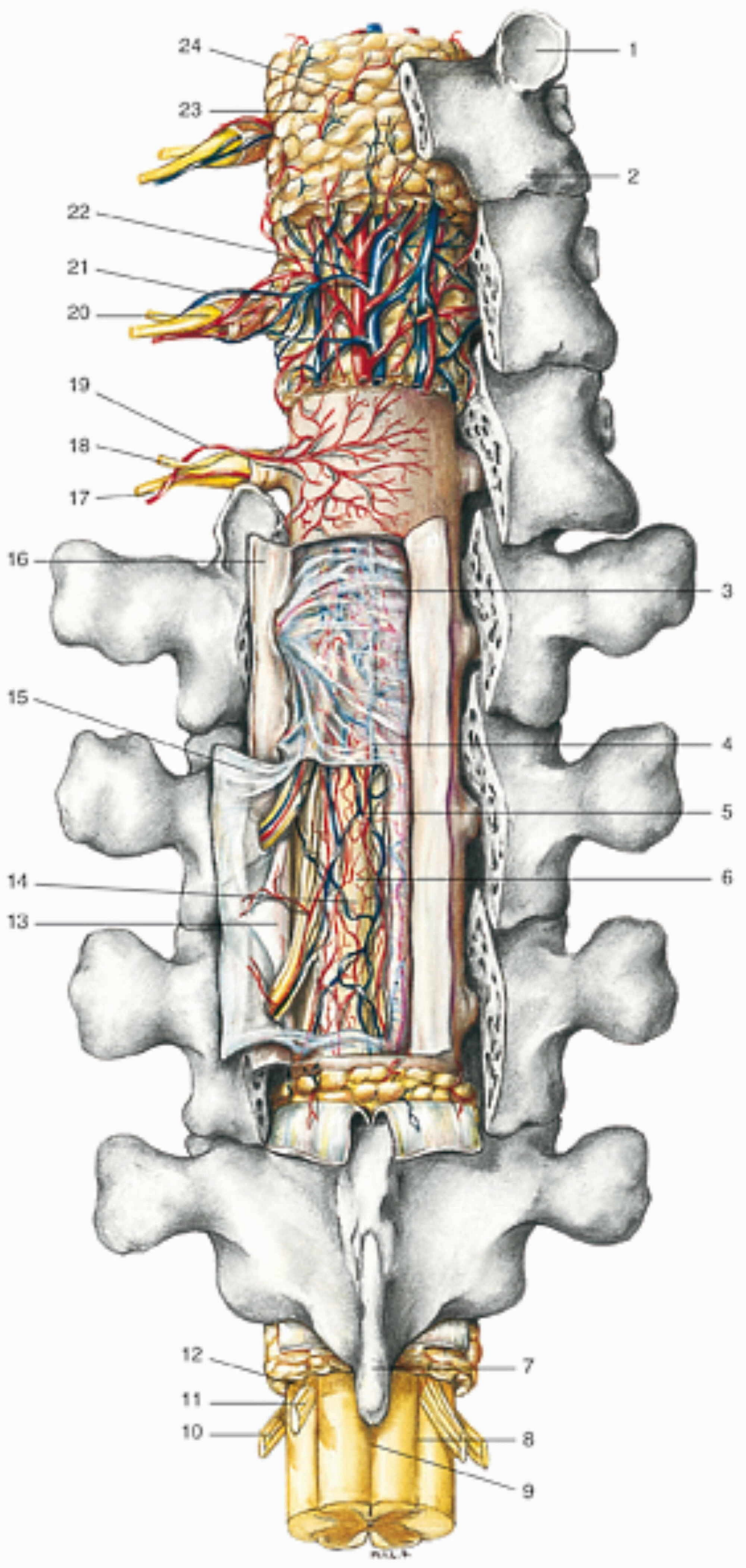
The figure highlights the structures of the spinal cord and the blood vessels, the meningeal layers, in a rear projection 1: upper joint process; 2: lower articular process; 3: subdural space; 4: rear septum; 5: posterior spinal vessels; 6: subarachnoid space; 7: spinous process; 8: posterior lateral sulcus; 9: median-posterior sulcus; 10: anterior motor root; 11: posterior sensory root; 12: interradicular septum; 13: denticulate ligament; 14: pia mater of spinal cord; 15: spinal arachnoid; 16: spinal dura; 17: anterior branch of the spinal nerve; 18: posterior branch of the spinal nerve; 19: spinal branch of the vertebral artery; 20: spinal ganglion; 21: spinal branch of the vertebral vein; 22: posterior vertebral venous plexus; 23: epidural fat; 24: epidural space. Reproduced with permission, from Anastasi G, et al., Anatomia dell’uomo, fourth edition [Human Anatomy], 2010, Milan: Edi-Ermes, Volume 3, p 188

In the cervical tract, we can find the sympathetic cervical chain, which runs over the longus muscles, from the longus capitis muscle to the longus colli muscle, passing under the prevertebral fascia [47]. The sympathetic upper cervical ganglion is located in the area of the C4 vertebra, with an oblong shape; the middle ganglion can reside either near the C5 or C6 vertebra (cricoid cartilage or cricoid tubercle) or C7, depending on the subjectivity of the individual [47]. The sympathetic system involves the districts of the tentorium and tongue and the cervical roots. It has been shown that in the ventral roots of all the medullary segments and in part in the axonic fibers, there are sympathetic cells identical to the cells that constitute the sympathetic ganglia of the corresponding anatomical chain [48]. We don't know the real function of these sympathetic cells. The sympathetic branches of the cervical tract innervate the spinal dura, both near the blood vessels and in areas without vessels [49]. The fibers of the sympathetic system are found within the vagus nerve for 3%-5% of the total, compared to all the parasympathetic fibers (Figure 3) [50].

## Conclusions

In this first part, I have highlighted the neurological relationships of the tentorium cerebelli, the musculature of the tongue, involving the pharyngeal plexus, the ansa cervicalis and the sympathetic cervical trunk, demonstrating the intimate relationships between the different body segments. In the second part, I will deal with the neurological interactions of the remaining diaphragms (thoracic outlet, diaphragm and pelvic floor) with clinical reflections. I will describe the phrenic and vagal relationships.
